# Incidence of the diagnosis of factitious disorders – Nationwide comparison study between Germany and Norway

**DOI:** 10.1007/s12024-020-00272-x

**Published:** 2020-06-11

**Authors:** Julian Geile, Jan Aasly, Burkhard Madea, Harald Schrader

**Affiliations:** 1 Institute of Legal Medicine, University Hospital Bonn, Bonn, Germany; 2 Department of Neuroscience, Faculty of Medicine, Norwegian University of Science and Technology (NTNU), Trondheim, Norway; 3 Department of Neurology, St. Olavs Hospital, University Hospital of Trondheim, Trondheim, Norway

**Keywords:** Factitious disorders, Illness deception, Munchausen syndrome, Incidence, Epidemiology

## Abstract

Factitious disorders (FD) like Munchausen syndrome are well known to most physicians, yet the corresponding ICD-10 diagnosis F68.1 remains severely under-assigned and often misdiagnosed. To approach this problem, we conducted a nationwide inquiry for Germany and Norway as well as a comparison between these two countries regarding the incidence of diagnosis of FD. The assignment rates of F68.1 in somatic hospitals from 2008 to 2016 were analyzed based on the Diagnosis Related Groups statistic from the German Federal Statistical Office and the data provided from the Norwegian Patient Registry. The Norwegian data also included information on individual patients whereas the German data only contained the total number of F68.1 assignment due to strict medical confidentiality laws. The incidence of the diagnosis of FD in Germany and Norway showed similar assignment rates with 3.71 and 3.18 per 100,000, respectively. The mean age was 39.4 years for German patients and 35.6 years for Norwegian patients. The gender distribution was almost equal for the individual patients’ rate (49% female and 51% male). Furthermore, our results indicate that female patients with FD tend to demand healthcare services more frequently than male patients. Smaller studies focusing on the diagnosis of FD have significantly higher assignment rates compared to nationwide inquiries. Our results illustrate substantial differences between estimations of the incidence of FD and the need for further studies. Besides the many obstacles associated with diagnosis of FD, strict medical confidentiality laws prevent reliable and scientific investigations of this matter.

## Introduction

Despite the fact that Munchausen syndrome [1] and other factitious disorders (FD) are well known to most physicians, the ICD-10 diagnosis F68.1 is severely under-assigned and misdiagnosed. The current Norwegian version of the ICD-10, like the English and German version, defines F68.1 as “intentional production or feigning of symptoms or disabilities, either physical or psychological”. Furthermore, it provides the following description: “The patient feigns symptoms repeatedly for no obvious reason and may even inflict self-harm in order to produce symptoms or signs. The motivation is obscure and presumably internal with the aim of adopting the sick role. The disorder is often combined with marked disorders of personality and relationships”. F68.1 includes “Munchausen syndrome”, “hospital hopper-syndrome” and “peregrinating patient” and excludes “factitial dermatitis” (L98.1) and “person feigning illness (with obvious motivation)” [2], i.e. Z76.5.

One of the reasons for FD being commonly under-assigned and misdiagnosed may be low awareness of the possibility of this disorder when confronted with an actual patient, fear of stigmatizing the patient with a pejorative connotation and concerns related to the possibility of reimbursement claims. In some cases, physicians will attempt to avoid further problems by discharging the patient as quickly as possible. A greater obstacle may be the effort involved in widening the investigation and reviewing previous diagnoses, especially when those have been made in other hospitals.

The result is enormous abuse and overuse of healthcare services and, not least, the risk of irreversible harm to these patients because of unnecessary invasive examinations and repeated interventions. In some cases, the injuries the patients incur as a result of medical treatment are greater than the injuries they inflict on themselves [3–5]. Therefore, FD may be associated with increased mortality [6].

The implementation of unnecessary diagnostic tests and intervention procedures also generates significant expenses. For the United States of America (US), the annual cost was estimated to be $40 million [7]. For Norway, it has been cautiously estimated that the cost for each patient with FD has averaged over one million Norwegian kroner ($90,000). One patient alone had costs of over six million Norwegian kroner ($540,000) [8]. There may also be negative consequences for people outside the hospital. For example, one of the most severe miscarriages of justice in post-war Germany occurred when an entirely innocent man was imprisoned for 5 years based on a false rape allegation by a woman with Munchausen behavior [9].

A different, emerging challenge regarding FD is the so-called “Munchausen by Internet”, first described by Feldman in 2000 [10]. It is considered a “virtual” FD in which the affected person presents a pattern similar to Munchausen syndrome on online-platforms like chat rooms, online support groups or social media. This pattern may include false representation of severe illnesses as well as dubious treatment recommendations, which in turn may inflict serious harm on other actual affected patients. Although there is no data on the incidence and harm caused by Munchausen by Internet, it can be assumed that it will be an increasing issue in the future, considering the continuously expanding range and usage of the World Wide Web.

Taking all these considerations into account, it seems pertinent to perform systematic studies on the magnitude of this issue. However, the vast majority of the 1200 PubMed publications on Munchausen syndrome, published after Sir Richard Asher first described this disorder in 1951 [1], are related to clinical presentation only. Very few large epidemiological studies have been performed, apart from the ones by Hamilton et al. [11] and Schrader et al. [12]. Both gave incidence numbers for the diagnosis of FD that had an entirely different order of magnitude than that found by earlier smaller, but carefully conducted studies by experienced physicians focused on FD [3, 4, 13–15]. Accordingly, there may be a great span of incidence numbers in different societies depending on the awareness of physicians and the willingness to assign the diagnosis of FD.

To address this issue, we performed a nationwide epidemiological study on the incidence of assignment of the ICD-10 diagnosis F68.1 in somatic hospitals in Germany and compared it with the findings in the nationwide study in Norway.

## Methods

### Germany

We used data from the German Federal Statistical Office (“Statistisches Bundesamt”) (StBA). The StBA is a federal authority of Germany, which reports to the Federal Ministry of the Interior. It collects, processes and analyses statistical information on economics, society, environment and health from all over Germany. For this study, the StBA provided data for the annual Diagnosis Related Groups (DRG) statistics. These statistics include every hospital that invoices based on the DRG compensation system, military hospitals that treat civilians, as well as hospitals of the German employer’s liability insurance association if the casualty or health insurance does not compensate the costs. Jail and police hospitals were excluded in these statistics, as well as psychiatric and psychosomatic institutions, since they use a different accounting system.

The DRG statistics were analyzed for the following variables regarding assignment of the ICD-10 diagnosis F68.1 from 2008 to 2016: total and mean number of assignments, mean age, gender distribution and the overall age interval. An assignment was defined as a single registered hospital case. Introduction of a diverse gender option for official documents in Germany occurred at the end of 2018. Therefore, no data on diverse genders were available. The individual patients’ rate could not have been determined since this data is anonymized by the individual hospitals before transmission to the StBA. The StBA examined the results for potential non-disclosure violations before final approval and resubmission. To calculate the assignment rate per 100,000 inhabitants we chose the German population of the median year (2012) of the examined period and divided the overall number of assignments by that population. For comparison with the Norwegian data, only annual assignment rates of the ICD-10 diagnosis F68.1 for all somatic hospitals from 2008 to 2016 were analyzed. Additionally, we were able to estimate the gender distribution and the mean age for each gender.

### Norway

Unpublished data from the nationwide Norwegian study on the incidence of diagnosis of Munchausen syndrome, other FD and malingering were analyzed from the Norwegian Patient Registry (NPR), a national institute that provides data for researchers and others seeking access to patient information. The Regional Committee for Medical and Health Research Ethics gave ethical approval. Data needed for the present study were available from 2008.

For comparison with the data provided by the StBA, we analyzed the assignment rates of F68.1 from 2008 to 2016. An assignment was defined as a single registered hospital case. The registry provided a de-identified list of all patients who had received the ICD-10 diagnosis F68.1 from 2008 to 2016. The information supplied by the NPR also contained a running number for tracking of each patient, year of birth, gender, year of contact with a healthcare provider and the name of the health institution, if it was within a somatic or psychiatric/psychological sector. It also included the name of one of the approximately 1700 specialists (somatic specialist, psychologist and/or psychiatrist) who have an operating agreement with one of the four Norwegian Regional Health authorities. Data were available about each hospitalization after which the diagnosis of F68.1 was given. In contrast to the data provided by the StBA, a specific running number for each patient allowed recording of the assignment rate as well as the individual patient rate. Detailed information about the latter appeared in the previous publication [12]. For the present comparison study, we excluded the psychiatric/psychological sector and data from specialists with operating agreements.

## Results

### Germany

A total of 2988 assignments with the ICD-10 diagnosis F68.1 were registered from 2008 to 2016 with an annual mean of 332 assignments (ranging between 236 and 465). Germany had approximately 80.5 million inhabitants in 2012 [16], resulting in a calculated assignment rate of 3.71 per 100,000 inhabitants. A total of 161,527,418 assignments were registered in the DRG statistics during the investigated period, meaning that F68.1 was diagnosed in approximately 0.0018% of all assignments. There was a considerable gender difference with 63% female and 37% male assignments as well as a difference regarding the mean age of those two genders (weighted mean of 38.1 years and 41.4 years, respectively). The weighted arithmetic mean age for all assignments and genders was 39.3 years.

### Norway

A total of 159 assignments with the diagnosis F68.1 were registered from 2008 to 2016. These corresponded to 78 individual patients, 48 females (61.5%) and 30 males (38.5%), giving an average number of hospital stays of 2.0 during this period. Norway had approximately 5 million inhabitants in 2012 [17], resulting in a calculated assignment rate of 3.18 per 100,000 and an individual patient rate of 1.56 per 100,000 inhabitants.

There was a significant gender difference with 77% females (123 of 159) and 23% males (36 of 159) based on the calculation from all assignments. The gender distribution calculated from all assignments was 49% for females and 51% for males with an average age for both genders of 35.6 years. Considering individual patients, female and male patients had an average age of 38.8 and 40.0 years, respectively. Table 1 shows a comparison between the German and Norwegian results.

**Table 1:** Comparison between the German and Norwegian results

	Germany	Norway
Total number of assignments	2988	159
Assignment rate (per 100,000)	3.71	3.18
Individual patients’ rate (per 100,000)	Not available	1.56
Gender distribution from assignments (f/m) (%)	63/37	77/23
Gender distribution of individual patients (f/m) (%)	Not available	49/51
Overall mean age from assignments (years)	39.3	35.6
Mean age (f/m) from assignments (years)	38.1/41.4	34.3/40.0
Mean age (f/m) of individual patients (years)	Not available	38.8/40.0
Average number of hospital stays	Not available	2.0

Nationwide assignment numbers and rates, gender distribution and mean age of patients with FD (ICD-10 diagnosis F68.1) from 2008 to 2016 in comparison between Norway and Germany. Assignment rates (registered hospital cases per 100,000 inhabitants) were calculated based on the 2012 inhabitant number of 5.0 million in Norway and 80.5 million in Germany. As patients may have more than one hospitalisation, the rates for assignments are higher than the rates for individual patients. The Norwegian results suggest that female patients with FD tend to demand healthcare services more frequently, since the gender distribution was almost equal for the individual patients’ rate

## Discussion

Systematic studies on the incidence of FD are relatively rare, with the major obstacle of obtaining a reliable and valid incidence being the nature of this disorder itself. The current data mainly consist of case reports and single case studies [18]. To the best of our knowledge, this is the first national inquiry regarding the incidence of diagnosis of FD in somatic hospitals for Germany and the first nationwide comparison study. The incidence of the diagnosis of FD in Germany and Norway showed similar assignment rates with 3.71 and 3.18 per 100,000, respectively. These results, as well as the results of the study by Hamilton et al. [11] from the National Hospital Discharge Survey of 400 to 500 general medical short stay inpatient hospitals (in which an assignment rate of 6.8 per 100,000 was found) had numbers that were significantly lower than those found in earlier much smaller studies.

The real incidence and distribution among the different medical specialties is still unknown and requires further studies. A systematic review of FD by Yates et al. concluded that patients with FD are most likely to present with endocrinological, cardiological and dermatological problems [19], whereas another review by Caselli et al. suggested that patients with FD are most frequently seen in psychiatry, neurology, emergency, and internal medicine departments [20]. Both reviews identified cases of FD in each major medical specialty. Yet, those results are based on case reports or case series and the choice of those reports implies a bias of publication and selection [20]. Furthermore, the variation may be explained by the particular interest among the authors working in different specialties [21]. Yates et al. assumed that the dermatologists’ increased awareness of or interest in FD may account for the high number of dermatology cases in their review “rather than a genuine ‘preference’ of patients with FD” [19]. This may also be due to the fact that dermatology represents a medical specialty that has included “factitial dermatitis” as a separate diagnosis for a FD for many decades [22].

It has to be acknowledged, that the identification of FD is complex, often time-consuming and requires systematic collection of relevant information, like a detailed chronology and thorough examination of the patient’s medical record. Ideally, the management of FD involves a team-based approach and close involvement of the primary care doctor [23]. The effort needed to identify a patient with FD may also notably differ depending on the medical specialty. The abuse of insulin in cases of factitious hypoglycemia may be comparably easy due to the possibility of detection in the laboratory [24, 25], whereas a patient presenting with factitious fever may require an extensive and multidisciplinary investigation. The review by Caselli et al. detected stressful or traumatic events in 20.2%, substance abuse in 16.9%, sexual abuses or neglect in childhood in 14.6%, suicidal behavior in 13.4%, conflicting and/or unstable interpersonal relationships in 10.7%, and premature familiar bereavements in 7.2% of patients with FD. Yet, a psychiatric consultation was refused by the patient or did not occur in about one third of the 514 identified cases [20]. These results illustrate that a laborious and sensitive consultation is needed to identify potential psychological risk factors. Aside from the lack of time many physicians are already facing in their daily routine, consultation refusal by the patients further interferes with successfully diagnosing FD. In conclusion, there is no reliable information about the distribution of FD patients among the medical departments to date and the results of systematic reviews of case reports and case series may indicate but do not reflect the real incidence and distribution.

Regarding previous publications on FD, Bauer and Bögner presented one of the most important early studies in 1996 [3] by carefully examining the hospital journals of 1538 patients in their neurological department in the hospital Charité, Berlin. These experienced physicians studied the number of patients with FD. They found five patients with FD (0.3%), four (0.26%) of whom were diagnosed with Munchausen.

Under the hypothetical assumption that the incidence of FD diagnosis in neurological departments is not significantly higher than in other departments, this corresponds to an individual patients’ rate of 325 per 100,000, i.e. 88 times more than that found for Germany in the present study. The Norwegian mean age (38.8 years for women and 40.0 years for men) of the patients was comparable with the results of the study by Bauer and Bögner (40.8 years) [3]. Although we were unable to determine the mean age for individual patients in Germany, the mean age based on the assignments was also similar (39.3 years).

Sutherland and Rodin made the diagnosis of FD in 10 of 1288 patients (0.8%) in a tertiary-care general hospital with a median age of 26 years (range from 19 to 64 years) and seven female and three male patients [13]. Another study by Kapfhammer et al. [14] identified 93 (76 females and 17 males) out of approximately 15,000 patients in the psychiatric consultation service of a university hospital during an 18-year-period, resulting in an incidence of 0.62%. The age of the female patients was 31.6 ± 4.2 years, and 31.3 ± 5.7 years for the male patients. Ferrara et al. identified 14 cases of FD (five females and nine males) in 751 patients in a Pediatric unit of a university hospital in Rome from November 2007 to March 2010, resulting in a prevalence of 1.8%. Munchausen syndrome was identified in three and Munchausen syndrome by proxy in four of the 751 patients. Age ranged between 11 months and 16 years with a mean age of 8.4 years [4]. Those numbers correspond to an individual patient rate of 1.864 per 100,000, i.e., 502 times more than the German assignment rate, and 1.194 times more than the Norwegian individual patients’ rate.

Table 2 shows a comparison of the calculated FD assignment per 100,000 inhabitants based on several studies. It is apparent that smaller studies that focused on the diagnosis of FD have significantly higher calculated assignments per 100,000 compared to nationwide inquiries.

**Table 2:** FD assignment per 100,000 based on several publications

Publication	Country	Investigated period	Source/Department	Total FD assignments / ratio	Calculated assignments per 100,000 inhabitants	Confirmed individual patients
Schrader et al.	Norway	2008–2016	Norwegian Patient Registry	159 assignments	3.18	Yes
Geile et al.	Germany	2008–2016	Nationwide DRG statistic	2988 assignments	3.71	No
Hamilton et al.	United States	1997–2006	National Hospital Discharge Survey	Not presented	6.8	No
Dahale et al. [26]	India	2001–2010	Neuropsychiatric center	8 of 81,176 patients	9.8	Yes
Bauer and Bögner	Germany	1992–1993	Neurology	5 of 1538 patients	325	Yes
Kapfhammer et al.	Germany	1978–1996	Psychiatric councils	93 of approx. 15,000 patients	620	Yes
Sutherland and Rodin	Canada	1985–1988	Tertiary-care general hospital	10 of 1288 patients	776	Yes
Ferrara et al.	Italy	2007–2010	Pediatric unit	14 of 751 patients	1864	Yes
Rumans and Vosti [15]	United States	1966–1976	Stanford University Medical Center	11 of 506 patients	2173	Yes

Studies investigation FD were compared and assignments per 100,000 inhabitants were calculated based on the total FD assignments or the presented ratio in the investigated period. Confirmed individual assignments meaning that each FD assignment could have been attributed to an individual patient

These results illustrate the difference between the estimations of the incidence of FD and the need for further studies. Furthermore, those differences indicate that FD are underdiagnosed in the daily clinical routine. A comprehensive nationwide evaluation of the incidence of FD, which also includes the data of psychiatric hospitals and primary care physicians, appears to be of value. On the one hand, the integrity of a patient has to be protected and it is important to prevent stigmatization of the patient. On the other hand, it has to be considered that patients with FD use up healthcare resources and may even harm other patients indirectly. Furthermore, patients with FD present an increased mortality and need to be protected from iatrogenic damage or death. The establishment of rigid criteria may help to reliably identify patients with FD, reduce the risk of stigmatization and ensure appropriate interaction with these patients during a potential confrontation.

## Limitations

The evaluation of the incidence of the diagnosis of FD contains several methodological challenges and pitfalls. Beside the already mentioned complexity and obstacles that accompany the successful diagnosis of FD in daily medical routine, this comparison study was limited by several factors. Data for the individual German patients (individual patients’ rate, gender ratio, mean age, and average number of hospital stays) could not be gathered due to strict German medical confidentiality laws. The number of assignments only allows an estimation of the real incidence and it remains unclear how many actual patients lie behind this number. This comparison study only included FD assignments in somatic hospitals in Germany and Norway. Further studies are required to gather information on the assignment rate and incidence for the psychiatric/psychological and primary care sector. Aside from underdiagnosing, misdiagnosing of F68.1 is another great problem in epidemiological studies of FD. In a sample of 24 patients, in which the original diagnosing healthcare providers undertook quality control measures after giving instructions, they found that the diagnosis was only correct in 11 patients [12]. Consequently, 54% of the patients were burdened with a negative diagnostic connotation and all its consequences. Related to this is the problem that arises from the removal of the diagnostic code of simulation with a clear motive (malingering, Z76.5) in the German ICD-10 modification. Although malingering represents one of the most important differential diagnoses to FD, it was removed in 1999 for reasons of “political correctness”, i.e. the avoidance of stigma for the patients. Lacking the correct diagnostic code for this type of simulation, the diagnosing physicians in the German part of the present study may have been tempted to use F68.1 instead of Z76.5. Further studies are urgently needed to figure out whether a significant misdiagnosis is also present in Germany. Of course, to fulfil the requirement of absolute anonymity of patients and to ensure that data cannot be traced back to the individual patient such studies represent a considerable methodological challenge. Another diagnosis within the scope of FD is the Munchausen by proxy syndrome (MBPS), which may be diagnosed under F68.1 as well as T74.8 or T74.9 (abuse). Further studies are needed to investigate which diagnosis MBPS is more commonly assigned to and if this results in a considerable statistical underrepresentation of FD. Taking all these issues into account, a revision of ICD-10 diagnosis regarding FD may be advisable to aid the physicians in correctly assigning FD. Further obstacles are revealed by the strict confidentiality laws that interfere with a more reliable and scientific inquiry on this matter, as shown by the German data. It is beyond dispute that data protection for patients, especially for those with FD, is of primary importance. Yet, the practical extent of this protection constitutes a serious problem for scientific work. It remains an unresolved ethical question and discussion is needed about whether the mere possibility of violating the integrity of an individual stands above the distributional justice of healthcare resources for the general population.

## Key points

- Factitious disorders remain under-assigned and often misdiagnosed.
- The incidence of the diagnosis of factitious disorders in Germany and Norway showed similar assignment rates with 3.71 and 3.18 per 100,000, respectively.
- The mean age was 39.4 years for German patients and 35.6 years for Norwegian patients.
- Our results indicate that female patients with factitious disorders tend to demand healthcare services more frequently than male patients.
- There are substantial differences between estimations of the incidence of factitious disorders.
